# Sustainable Export Innovation Behavior of Firms Under Fiscal Incentive

**DOI:** 10.3389/fpsyg.2021.769795

**Published:** 2021-11-04

**Authors:** Chen Feng, Beibei Shi, Hong Yan, Siying Yang, Caiquan Bai

**Affiliations:** ^1^School of Public Economics and Administration, Shanghai University of Finance and Economics, Shanghai, China; ^2^School of Economics and Management, Northwest University, Xi'an, China; ^3^School of International Relations and Public Affairs, Fudan University, Shanghai, China; ^4^Center for China Public Sector Economy Research, School of Economics, Jilin University, Changchun, China; ^5^The Center for Economic Research, Shandong University, Jinan, China

**Keywords:** fiscal centralization, sustainable export innovation behavior, income tax sharing reform, China, firm

## Abstract

The fiscal imbalance between the central and local governments under fiscal centralization may motivate local governments to pass tax burdens on firms. The causal identification of the tax system reform and the sustainable export innovation behavior of firms are of great significance. This study uses the income tax sharing policy of China to examine the impact of fiscal centralization on the sustainable export innovation behavior of firms. We find that this tax reform has significantly inhibited the increase of the export value-added rate of firms, and has an increasing trend with the share ratio between the Central Government and the local government. Moreover, this effect mainly comes from the crowding-out effect of imported intermediate goods on domestic intermediate goods. The tests show that the above conclusions are consistent with the general logic of local governments. When they face greater downward fiscal pressure, they will further pass the tax burden on local firms and force the firms to promote their export performance to expand the tax base. This short-sighted behavior of replacing “quality improvement” with “quantity increase” is an important factor that affects the sustainable export innovation behavior of firms and the climb in the global value chain.

## Introduction

The sustainable development of firms has always been an important topic, both in theoretical research and firm practice. In particular, the government plays a crucial role. How do the macro policies (i.e., fiscal policies) of a government affect sustainable innovation behavior in exporting firms? In the past decades, China has increasingly participated in the global value chain and become an important manufacturing center and export platform in global production networks. As the largest exporter in the world, China has gained its reputation as a “world factory.” However, China has long been locked into an inherent label of low domestic value-added rate (DVAR) in exports, “made in China” rather than “created in China” in the international market. In the context of current global market consolidation and international trade frictions, the discussion on how to improve the sustainable export innovation behavior of Chinese firms is of great importance. Since the 1990s, the international division of labor has undergone important changes. Specifically, the transfer of processing mode from the inter-industry to intra-industry division of labor has gradually become the mainstream mode of international production and trade development. With the deepening of the international division of labor, the production value-added links of firms (including design, R and D, manufacturing, assembly, and marketing, etc.) and the rise of global value chains have gradually become important targets of widespread attention by researchers in the industrial sector and academia (Mao and Xu, 2018). At present, most of the literature uses DVAR to represent the degree of sustainable development of domestic export products and reflect the sustainable export innovation ability in the global value chain (Koopman et al., 2012; Upward et al., 2013). For firms, the improvement of DVAR is conductive to obtaining more real trade gains, and it is also an important part of the sustainable innovation behavior of a firm. Therefore, the study on the DVAR of export firms is of great theoretical value and practical significance to understand the sustainable export innovation behavior of firms.

Since the financial crisis broke out in 2008, fiscal centralization has been a common trend across different countries in the world. The reform of income tax revenue sharing in China provides an opportunity for us to explore the impact of fiscal centralization on the sustainable export innovation behavior of firms. The implementation of a scheme on the reform of income tax revenue sharing on January 1, 2002 by the Chinese government is a typical manifestation of centralization of central fiscal. In this reform, firm income taxes shifted from local taxes to the taxes shared by the central and local governments. The proportion of revenues of local governments in the collection of income tax revenues dropped sharply from 50 to 100%, and further dropped to 40% in 2003. It can be seen that tax reforms are important measures in the history of taxation. However, what surprised us is that the existing research pays little attention to this fiscal centralization, and the only research focuses on corporate tax evasion (Fan and Tian, 2013; Tian and Fan, 2016), local tax transfer (Han and Kung, 2015), average labor added value (Li et al., 2018), and firm productivity (Cai et al., 2018), etc.

In this study, this reform is used as a quasi-natural experiment to make a causal inference between fiscal centralization and the DVAR of firms. It is found that for those firms affected by the reform in the “intervention group,” their DVAR decreased significantly after tax sharing, compared with the export firms in the “control group” that pay income tax to the state taxation bureau. The above results are still valid after a set of robustness tests and the consideration of macroeconomic environment. Additionally, by analyzing the influence mechanism, we find that the real cause of the decline in DVAR is not related to the variation of cost markup rate, but mainly the crowding-out effect of the intermediate goods trade under fiscal centralization. However, such a crowding-out effect has a greater impact on processing trade firms that have greater elasticity to the price of intermediate goods but has an insignificant impact on the general trade firms and mixed trade firms that have relatively small elasticity. Additionally, for the local governments, in order to improve their own tax revenue under great fiscal pressure, they will put forward requirements for the export performance of firms under their jurisdiction. At the expense of the decline in DVAR and export investment of local firms, industrial output and total export volume will be promoted. Such a policy guidance mode of “quality” for “quantity” will have a negative impact on the long-term sustainable development and export innovation behavior of firms.

The marginal contribution of this study may be reflected in the following: first, the intentional consequences and related impacts of fiscal and tax centralization are still inconclusive in academia. This study focuses on micro firm data to explore the effect of fiscal policy on the sustainable innovation behavior of firm export, providing a clear explanation and mechanism analysis to some certain extent. Second, the implementation of fiscal policies tends to be highly endogenous, and the analysis of the economic effects of macro tax rate fluctuations is inevitably influenced by the bias of unobvious omitted variables (Romer and Romer, 2010; Han and Kung, 2015; Li et al., 2018). Based on the exogenous shock of the reform, we construct a difference-in-difference (DID) model using the difference in ownership when export firms are paying taxes. The causal mechanism is identified by the different times of establishment of firms, so as to obtain the “net effect” of fiscal policy more accurately. Third, due to data limitation, previous empirical studies on the sustainable export behavior of Chinese micro firms often combine customs data with the China industry business performance data from 2000 to 2007 (Upward et al., 2013; Zhang et al., 2013; Kee and Tang, 2016; Mao and Xu, 2018). However, at the beginning of the new century, international and domestic economic and political policies changed frequently, many major policies and macro environments, such as the accession of China to the World Trade Organization (WTO), reform in value-added tax (VAT), changes in export tax rebate regulations, and the economic cycle may have an influence on the empirical results. These factors have not been fully considered in the previous study. Therefore, in this study, a set of robustness tests is used to check the potential factors that may cause estimation errors one by one to make a solid confirmation of the final results.

The rest of this article is organized as follows: Section Policy Background and Theoretical Hypothesis introduces policy background and theoretical hypothesis. Section Model Setting, Indicator Calculation, and Data Sources presents the models, indicator calculation, and data. Section Empirical Results and Analysis discusses the results of the empirical and robustness tests. Section Influencing Mechanism explores the mechanism of influence. The last section draws the Conclusions.

## Policy Background and Theoretical Hypothesis

### Policy Background

Fiscal and tax policies are important measures taken by the government to implement national governance and optimize resource allocation, which is essentially a special distribution relationship formed by the state in participating distribution and redistribution of national income, so as to realize its functions. Because of financial difficulties and planned economy, the financial budget management system was dominated by centralized arrangements for revenue and expenditure. However, it has strictly restricted the independent right of local taxation. After the reform and opening up, in order to promote the coordinated development of the central and local governments and achieve the core goal of economic growth, the Fiscal Responsibility System came into being. Compared with the previous situation where local governments had no tax autonomy, under the Fiscal Responsibility System, local governments pay a fixed amount of fiscal revenue to the central government and have the right to make budgets and claim the surplus from their autonomous tax revenue (Chen and Gao, 2012; Tian and Fan, 2016). The excessive tax revenue is completely controlled by the local government, which greatly improves the decentralization and autonomy of local tax management. It is not only conducive to the improvement of the tax efforts by local government but also provides incentives for the growth of the tax revenue within the budget. However, such tax incentive can easily lead to the moral hazard of local governments. Hiding the tax sources, reducing the proportion of central tax sharing, and seeking subsidies with low tax declaration will be the “rational” choice of local governments under this system.

Under the pressure of the fiscal crisis, the central government launched the tax-sharing reform at the beginning of 1994. This reform aimed to re-establish the economic management power of the central government over important taxation areas (Zhang, 2008; Chen and Gao, 2012). In this case, the trend of fiscal centralization gradually emerged. On the one hand, the central government has clearly defined the boundaries among the revenue of central tax, local tax, and shared tax. On the other hand, the central government has also begun to tighten its grip on local taxes and shared taxes. In this context, the reform of income tax revenue sharing came into being.

On December 31, 2001, the State Council issued the *Circular of the State Council on Distributing the Scheme on the Reform of Income Tax Revenue Sharing* (No. 37 [2001] of the State Council), which decided to implement the reform in the sharing of revenue from income tax from January 1, 2002 and also regulated the proportion for the central and local governments to share the revenue from income tax. In accordance with the provisions of the *Circular*, the income tax payments of railway transport firms, state-owned post firms, Industrial and Commercial Bank of China Limited, Agricultural Bank of China, Bank of China Ltd., China Construction Bank Corporation, China Development Bank, Agricultural Development Bank of China, Export-Import Bank of China, and China Petroleum and Chemical Corporation, as well as offshore oil and natural gas firms continue to be the revenue of the central government. Other firm income tax and individual income tax are shared by the central and local governments in proportion. Since 2002, the proportion to share revenue from income taxes shall be 50% for the central government and 50% for the local government. Since 2003, the proportion to share revenue from income taxes shall be 60% for the central government and 40% for the local government.

In addition, in order to prevent the dislocation of tax collection and management, after the launch of the reform, the scope of firm income tax collected and managed by the state and local administration of taxation and shall not be changed before 2002. It can be seen that this system is an important measure for the reform of fiscal and tax relations between the central and local governments. The income tax of newly registered firms and institutions will be collected uniformly by the State Administration of Taxation since the implementation of the reform. The implementation of this reform provides the conditions for us to construct a DID model. In accordance with the *Circular*, firms that are not the Chinese central state-owned established after 2002 shall pay income tax to the Local Administration of Taxation instead of the State Administration of Taxation. However, the income tax of Chinese central state-owned firms and foreign-funded firms continues to be paid to the State Administration of Taxation, which is not influenced by the reform. This means that firms of the same type need to face completely different tax collection administrations because of differences in establishment time. Especially for old firms established before 2002, although the tax collection administration has not changed, the decline in the tax share ratio may also cause them to face completely different taxation efforts before and after the reform. Therefore, we can regard the Chinese central state-owned firms and foreign-funded firms whose income taxation are collected by the State Administration of Taxation as the control group, and the other types of firms established before 2002 as the intervention group (Tian and Fan, 2016), so as to employ the DID method to estimate the impact of the reform of income tax revenue sharing on the DVAR of export firms.

### Theoretical Hypothesis

Under the reform of income tax revenue sharing, the grabbing hand of the central government is likely to cause the moral hazard of the local government while dividing and sharing the local taxes, which will have an important impact on the behavior of local firms. The logic behind it is as follows: first, tax sharing will cause the effect of tax burden shifting to local firms. The grip of the central government on local government tax revenue will not only reduce local fiscal revenue but also urge it to exert efforts on local economic performance to make up for the loss. As the main participants in the market, firms are the only choice to afford the tax burden. Han and Kung (2015) find that the reform of income tax revenue sharing will induce local governments to vigorously develop real estate, commerce, and industrial undertakings to make up for the fiscal deficit caused by the decrease in income tax. Second, tax sharing will encourage firms to evade taxes, which is because of the decline in tax law enforcement and tax collection efforts of local governments in attracting investment (Fan and Tian, 2013; Tian and Fan, 2016). To attract the inflow of firm liquidity elements and compete for the income tax base, local governments will have the incentive to reduce the intensity of tax collection and management and help firms to evade tax in the competition of tax sources. Meanwhile, evading tax will significantly reduce the effective tax rate to be paid by firms (Li et al., 2018) as well as corresponding costs and capital constraints of firms (Cai et al., 2018). Therefore, in the dual roles of being “squeezed” and “flattered,” firms enjoy the bonus of reducing cost brought by lightening the tax burden on the one hand, and on the other hand, they have to undertake more responsibilities of production and transformation. We assume that it is the two-way distortion of firm behavior that has a significant impact on its DVAR and, thus, sustainable export innovation behavior.

When the cost of production and operation of a firm is reduced, its export advantages are highlighted. Lowering the entry barrier to the foreign trade market provides an additional profit space for more firms to participate in the market, so firms with lower productivity can also participate in exporting. In this way, the average productivity of exporting firms will decrease (Yu and Cui, 2018), while the relative competitiveness of incumbent firms will be improved. The increase in the cost markup rate of firms will thus further promote the export value-added rate (Berman et al., 2012). However, compared with domestic-sale firms, export firms whose main business is the export of products and services will undertake more responsibilities to promote export performance after local tax sharing. The two incentives, namely the downward pressure on local fiscal deficit and the increase in profit margin, will further encourage the firms to increase the export and investment demand for intermediate goods. Compared with the constant price of imported intermediate goods, the increase in demand for intermediate goods will inevitably generate an incentive to raise their price. Therefore, the relative price of imported intermediate products will be reduced. Imported intermediate goods, thus, have a crowding-out effect on the market input of domestic intermediate goods, which will further inhibit the increase in the DVAR of firms.

Overall, on the one hand, the DVAR of firms will rise because of the cost markup effect when the central government participates in tax sharing. On the other hand, it will reduce because of the crowding-out effect of intermediate goods. Then, what is the overall effect of the fiscal centralization on the DVAR of firms? We will perform an empirical analysis to investigate the question. Additionally, empirical tests are carried out to examine the two influencing mechanisms.

## Model Setting, Indicator Calculation, and Data Sources

### Model Setting

To investigate the effect of tax sharing on the DVAR of firms, this study uses the tax reform by the central government for firms that are not Chinese central state-owned or foreign-funded in 2002 as a quasi-natural experiment, and uses the DID method to evaluate the causal effect between tax sharing and DVAR of firms. We obtain the “net effect” by comparing the change in the DVAR of different firms before and after the tax sharing reform. Therefore, the following model is established:


(1) $$\begin{array}{l} DVAR_{it}=\alpha +\beta Share_{i}\times Post_{t}+\sum Control_{it}+\mu_{i}+\delta_{t}+\varepsilon_{it} \end{array}$$

where *DVAR*_*it*_ represents the export value added-rate of the firm *I* in the year *t*; *Share*_*i*_ represents the firm affected by the tax sharing reform. If a firm is non-state-owned and non-foreign established before 2002, the value of this indicator is 1, otherwise, the value is 0; *Post*_*t*_ is the time dummy variable of tax sharing reform, which is 1 after 2002 and 0 before 2002; *Control* represents a set of control variables that affect the export value-added rate of the firm; μ_*i*_ and δ_*t*_ is the firm fixed effect and the year fixed effect respectively; ε_*it*_ is a random error term. β is used to measure the effect of tax sharing on the DVAR of firms. If β < 0, it means that tax sharing reduces the DVAR of firms; otherwise, it increases the DVAR of firms. In addition, the standard errors are basically clustered at the firm level.

Of course, model (1) only estimates the average treatment effect of tax sharing reform on the DVAR. In fact, considering the lag of policy implementation, information transmission and firm strategy adjustment, and other factors, tax sharing is not certainly effective in the current period, and the effect of the reform may be delayed and long-term. Based on the above reasons, this study extends model (1) and establishes a dynamic effect model as follows:


(2) $$\begin{array}{ll} DVAR_{it}=\theta +\underset{\tau \in \left\{2002,2003,\cdots \;,2006\right\}}{\sum }\theta_{\tau }Share_{i}\times Post_{\tau }\\ + & \sum Control_{it}+\mu_{i}+\delta_{t}+\varepsilon_{it} \end{array}$$

where *Post*_τ_ represents the year dummy variable of τ years after tax sharing reform implementation (where τ ∈ {2002, 2003, ⋯, 2006}). θ_τ_ measures the dynamic effect of tax sharing on the DVAR of firms. The description of other variables is the same as the model (1).

### Calculation of DAVR of Firms

For the calculation of added-value rate, Hummels et al. (2001) first proposed the Hummels-Ishii-Yi (HIY) method to calculate foreign value-added ratio (FVAR). With the further expansion of research, scholars began to pay more attention to the domestic value-added ratio (DVAR). According to different measurement objects, the methods can be divided into macro-level and micro-level measurement methods. At the macro level, the input-output table is used to calculate the DVAR. The biggest advantage of this method is that it does not need to assume the share of foreign materials in domestic raw materials in advance, and that imported intermediate goods can also include service input. At the micro level, the temporal trend of DVAR can be investigated more comprehensively by this calculation method, which can deepen the analysis of the heterogeneity of firms. Considering the micro-level measurement method can better investigate the impact of exogenous factors on DVAR (Zhang et al., 2013), the wide existence of firm heterogeneity (Melitz, 2003), and for the content of this study, the micro-level measurement method is adopted to measure the DVAR of firms.

Following Upward et al. (2013), Zhang et al. (2013), and Kee and Tang (2016), this study uses the China Industry Business Performance Database and China Customs Trade database to calculate the DVAR of firms, which can be expressed as follows:


(3) $$\begin{array}{l} DVAR_{ijt}=\left(EX_{ijt}^{O}+EX_{ijt}^{P}\right)-\left(\frac{EX_{ijt}^{O}+EX_{ijt}^{P}}{Y_{ijt}}\right)\times \left(IM_{ijt}^{IO}+IM_{ijt}^{IP}\right) \end{array}$$

where *i, j*, and *t* represent firm, industry, and time, respectively. $EX_{ijt}^{O}$ and $EX_{ijt}^{P}$ represent the general export and the processed export, respectively. $EX_{ijt}^{O}+EX_{ijt}^{P}$ represents the total export of the firm, which can be obtained from the customs trade database. $IM_{ijt}^{IO}$ and $IM_{ijt}^{IP}$ represent the general import of intermediate goods and the processed import of intermediate goods, respectively. $IM_{ijt}^{IO}+IM_{ijt}^{IP}$ measures the total import of intermediate goods by the firms. *Y*_*ijt*_ is the output of firms, which is measured by the gross value of industrial output by the firms. It can be obtained directly from the China Industry Business Performance Database.

The current problem is how to obtain the total import of intermediate goods by firms. Since the indicator cannot be obtained directly, it needs to be obtained indirectly. In order to obtain it more accurately, this study explains the relevant problems in the process of treatment: (1) in the sample period, since most Chinese firms rely on the intermediate merchants to import and export, it is necessary to identify the trade intermediaries. Following the previous method, firms whose names include “economy and trade,” “science and trade,” “import and export,” “foreign trade,” and “trade” in the China Custom trade database are defined as trade intermediaries in this study, and the actual import of intermediate goods is adjusted (Ahn et al., 2011); (2) match the BEC product code of the United Nations and product classification code of China customs to identify the intermediate goods, capital goods, and consumption goods included in the imported goods; (3) the foreign content share that Chinese firms use in the input of their domestic intermediate goods is around 5–10% (Koopman et al., 2012). According to the previous research, we assume that the foreign content share in domestic raw material is 5%^1^; (4) the existence of trade agents makes firms over import, so the observed value of over import phenomenon is eliminated in this study^2^. Similarly, each index has been *Winsorized* at the 1% level.

After the above preliminary treatments, and combined with the difference in the mode of intra-firm trade, the calculation formula of DVAR can be further expressed as follows:


(4) $$\begin{array}{l} DVAR_{ijt}=\\ \{\begin{array}{c} 1-\frac{{IM_{ijt}^{R\_O}|}_{BEC}+IM_{ijt}^{F}}{Y_{ijt}},i\in O\\ 1-\frac{{IM_{ijt}^{R\_P}|}_{BEC}+IM_{ijt}^{F}}{Y_{ijt}},i\in P\\ \Theta_{O}\times (1-\frac{{IM_{ijt}^{R\_O}|}_{BEC}+IM_{ijt}^{F}}{Y_{ijt}})+\Theta_{P}\times (1-\frac{{IM_{ijt}^{R\_P}|}_{BEC}+IM_{ijt}^{F}}{Y_{ijt}}),i\in M \end{array} \end{array}$$

where *O, P*, and *M* represent general trading firms, pure processing trading firms, and mixed trading firms, respectively^3^. The same assumptions as the previous research are adopted: various raw materials used by firms to produce domestic sales products and exported products are the same. $\;IM_{ijt}^{R\text{\_}O}{|}_{BEC}$ represents the actual volume of intermediate goods imported by the general trading firm *i* at period *t*. $IM_{ijt}^{R\text{\_}P}$ represents the actual volume of intermediate goods imported by the pure processing trading firm *i* at period *t*. $IM_{ijt}^{F}$ represents the foreign content share in the domestic raw materials used by firm *i* in the period *t*. Θ_*O*_ and Θ_*P*_ represents the share of intermediate goods imported by mixed trading firms in the general trade and pure processing trade.

### Selection of Control Variables

In addition to the core explanatory variable, there are many other factors that may influence the DVAR of firms. Therefore, following the relevant literature, this study selects the productivity, age, and market competition of firm as control variables to control the influence of relevant factors, so as to evaluate the net effect of tax sharing on the DVAR of firms. Specifically, the control variables are measured as follows:

(1) Firm age. It is represented by the logarithm of 1 plus the difference value between the year of the current year and the year when the firm started business. (2) Total firm profit. It is expressed by the logarithm of the total profit of firms (10,000 Yuan). (3) Firm scale. It is represented by the logarithm of the total assets of firms. (4) Firm capital intensity. It is measured by the ratio of net fixed assets to the average number of employees in the firm (logarithmic value). (5) Firm export capacity. It is represented by the logarithm of the total export volume of the firm. The more the total exports, the stronger the export capacity of firms, otherwise, the weaker the export capacity. (6) Firm productivity. There are several existing methods to calculate the productivity of firms, such as ordinary least squares (OLS) regression, fixed effects (FE) model, Olley-Pakes (OP) method, Levinsohn-Petrin (LP) method, and Generalized Method of Moments (GMM). Generally, there is a great controversy in calculating the productivity of micro firms with the OLS and FE methods, since they are not effective in solving endogenous bias. Following Yang (2015), the OP method overcomes the endogenous bias and selective bias, so it is used to calculate firm productivity in this study. (7) Weighted average import tariff of firm (*Tariff*). It is used to measure tariff level faced by firms when they import. The specific calculation method is as follows:


(5) $$\begin{array}{l} Tariff_{it}=\log\left(\frac{\sum_{n=1}^{m}Aerage\;Rate^{HS6}\times Value_{it}^{HS6,n}}{Total\;Value_{it}}\right) \end{array}$$

where *Tariff*_*it*_ represents the weighted average import tariff of the firm *i* in the year *t*. Assume that the firm *i* imports a total of *m* kinds of goods in the period *t*, *Aerage Rate*^*HS*6^ represents the average import tariff of the goods under the first 6-digit HS code of the corresponding customs. $Value_{it}^{HS6,n}$ represents the value of the *n*-th kinds of goods under the first 6-digit HS code of the import of firms in the period *t*. *Total Value*_*it*_ represents the total import of the firm *i* in the period *t*. The average import tariff rate of goods under the first 6-digit HS codes of the customs is obtained from the WTO website. However, since the data of the import tariff of China in 2000 is not published on the WTO website, and considering that China has not substantially reduced its import tariff from 1997 to 2000 (Yu, 2011), this study uses the arithmetic mean value of the tariff in 1996 and 1997 to measure the level of import tariff in 2000. (8) Herfindahl-Hirschman Index (HHI). This study uses the HHI of firms in the binary industry to measure the degree of market competition faced by firms. The specific measurement method is as follows:


(6) $$\begin{array}{l} HHI_{jt}=\underset{i\in j}{\sum }{\left(scale_{it}/scale_{jt}\right)}^{2}=\underset{i\in j}{\sum }{\left(S_{it}\right)}^{2} \end{array}$$

where *scale*_*it*_ is the scale of firm *i* in year *t*, which is measured by sales volume. *scale*_*jt*_ is the scale of industry *j* in year *t*, which is measured by total sales volume of the industry. *S*_*it*_ is the market share of the firm *i* in the year *t*. The larger the index is, the higher the degree of market monopoly is; otherwise, the higher degree of market competition is. Meanwhile, we also select variables, such as regional gross domestic product (GDP), government fiscal expenditure, and urbanization level, to measure the main economic factors at the city level. The city factors are controlled below to prevent them from affecting the results of this article. Finally, by matching between major databases, a total of 56,153 firm-level observations are obtained. The descriptive statistics of the specific variables are shown in Table 1^4^.

**Table 1:** Descriptive statistics of variables.

**Variable**	**Definition**	**Mean**	**Std. dev**.	**Min**.	**Max**.
DVAR	Domestic value-added ratio	0.8026	0.1938	0.0041	0.9987
*Share*	Firm affected by the reform	0.2944	0.4558	0	1
*Middle*	Firm intermediate goods	6.8875	1.6913	1.9692	15.6950
*Mkp*	Domestic cost markup rate offirm	1.1927	0.0978	0.9041	2.3831
*TFP*	Firm productivity	6.5295	1.0037	2.6077	10.1619
*HHI*	Herfindahl-Hirschman index	0.0450	0.7986	0	68.5782
*density*	Firm capital intensity	0.9629	2.1146	0.0001	126.9433
*Tariff*	Weighted average import tariff offirm	3.6864	0.6581	0	5.5620
*export*	Firm export capacity	0.9986	1.6712	−9.2103	9.6291
*age*	Firmage	2.2981	0.4829	1.6094	4.0604
*profit*	Total profit offirm	5.3941	1.9796	−1.6094	14.4191
*size*	Firm scale	10.9179	1.4840	5.3279	18.7282
*gdp*	Logarithm of GDP	9.0612	0.7657	4.7690	10.1720
*open*	FDI/GDP	81.4248	63.2174	0.0066	222.3713
*government*	Government fiscal expenditure/GDP	0.1308	0.0530	0.0691	0.8507
*financial*	Total regional loans/GDP	0.7701	0.5611	0.0001	2.2522
*urban*	Total urban population/Total population	0.4952	0.1878	0.0045	0.8870
*wage*	Logarithm of total urban salary	9.7934	0.3396	8.7819	10.5996

### Data Sources

Based on the data from China Industry Business Performance Database and China Customs Trade database from 2000 to 2006, this study mainly evaluates the impact of tax sharing on the DVAR of firms from the micro firm level. We match the data from the two databases in detail, taking the matched samples as the database of analysis. Of course, before matching, we treat the data as follows:

For the China Industry Business Performance Database, we make the following preliminary selections for the original data. The following samples are excluded: (1) non-operating firms; (2) negative or missing values of the variables (total assets of firms, net fixed assets, sales volume, total industrial output, and industrial added value) we used in the process of analyzing; (3) firms with an average number of employees <8; (4) firms that are not Chinese central state-owned and with a sales volume >5 million yuan; (5) firms with a profit margin lower than 0.1% or higher than 99%. In addition, according to Generally Accepted Accounting Principles (GAAP), the following samples are also excluded: (1) firms with current assets greater than total assets; (2) firms with fixed assets greater than total assets; (3) firms with net fixed assets greater than total assets; (4) firms with invalid establishment time; (5) firms with accumulated depreciation less than the depreciation. After the above preliminary treatment, the samples were *Winsorized* at the 1% level.

According to the issue to be studied in this article, we need to merge the treated data from the China Industry Business Performance Database and China Customs Trade database as much as possible. Following Yu (2015), the specific steps of merging the data mainly are as follows: the first step is to match the industry business performance data and customs data of the same year according to the name of firms; the second step is to match the unsuccessfully matched firm samples according to the post code and the last 7 digits of the phone number; the third step is to merge the two matched results. The samples after the above matching are the basis of the data of this study. Of course, in addition to the micro firm data, in order to make the analysis more detailed, the import tariff data of China from 1996 to 2006 is also used, which is obtained from the Tariff Download Facility Database of WTO. Meanwhile, the product and industry codes are used frequently in this study, so the classification by Broad Economic Categories (BEC) and Harmonized System (HS) customs code conversion table is selected, which is obtained from the United Nations website. In the section of robustness tests, different macro policies and economic factors are excluded. The relevant policies are from government documents, and the economic indicators are from *China Statistical Yearbook* (2000–2006).

## Empirical Results and Analysis

### Impact of Tax Sharing on DVAR of Firms

This study mainly investigates the impact of the tax sharing reform implemented by the central government in 2002 on the DVAR of firms, which can be estimated by model (1). The specific results are shown in columns (1) and (2) in Table 2. In column (1), only the impact of tax sharing on the DVAR of firms is investigated. The results show that the tax sharing reform has a negative effect on the DVAR, and preliminarily indicate that tax sharing is not conducive to improving the DVAR of firms. After controlling firm fixed effect, time fixed effect, and other related factors, the results are shown in column (2). The results show that after the control of other factors, tax sharing reduces the DVAR of firms, and that the effect is significant at the level of 1%. In addition, considering the particularity of Shanghai, it has been implementing tax sharing between the central and local governments for the non-central state-owned or non-foreign-funded firms. Therefore, the firms in Shanghai are not be influenced by the tax sharing reform in 2002, which causes the bias of the evaluation in this study. Considering this particularity, we exclude all the firms in Shanghai and re-estimate the model (1). The results are shown in columns (3) and (4) in Table 2. It can be seen that the exclusion of firms in Shanghai does not influence the impact of tax sharing reform on the DVAR of firms. Additionally, compared with columns (1) and (2) in Table 2, the effect of tax sharing reform has enhanced, which indicates that the inclusion of firms in Shanghai leads to the underestimation of the effect of tax sharing reform on the DVAR of firms.

**Table 2:** Impact of tax sharing reform on the domestic value-added rate (DVAR) of firms.

	**DVAR**	**DVAR**	**DVAR**	**DVAR**	**DVAR**	**DVAR**	**DVAR**	**DVAR**
	**(1)**	**(2)**	**(3)**	**(4)**	**(5)**	**(6)**	**(7)**	**(8)**
*Share × Post*	−0.0209^***^	−0.0187^***^	−0.0214^***^	−0.0189^***^				
	(0.0023)	(0.0023)	(0.0025)	(0.0024)				
*ShareRatio × Post*					−0.0050^*^	−0.0043^*^		
					(0.0026)	(0.0026)		
*Share × Post2002*							−0.0035	−0.0026
							(0.0029)	(0.0029)
*Share × Post2003*							−0.0083^***^	−0.0072^**^
							(0.0029)	(0.0029)
*Share × Post2004*							−0.0119^***^	−0.0090^***^
							(0.0031)	(0.0031)
*Share × Post2005*							−0.0333^***^	−0.0314^***^
							(0.0031)	(0.0031)
*Share × Post2006*							−0.0490^***^	−0.0454^***^
							(0.0034)	(0.0035)
*TFP*		0.0397^***^		0.0399^***^		0.0397^***^		0.0396^***^
		(0.0017)		(0.0019)		(0.0017)		(0.0017)
*HHI*		−0.0001		−0.0006		−0.0002		−0.0002
		(0.0014)		(0.0028)		(0.0014)		(0.0014)
*density*		−0.0010^*^		−0.0016^**^		−0.0010^*^		−0.0008
		(0.0006)		(0.0007)		(0.0006)		(0.0006)
*Tariff*		0.0060^***^		0.0066^***^		0.0061^***^		0.0062^***^
		(0.0017)		(0.0018)		(0.0017)		(0.0017)
*export*		−0.0082^***^		−0.0088^***^		−0.0084^***^		−0.0079^***^
		(0.0011)		(0.0013)		(0.0011)		(0.0011)
*age*		0.0091^***^		0.0091^***^		0.0117^***^		0.0072^**^
		(0.0030)		(0.0030)		(0.0030)		(0.0029)
*profit*		−0.0030^***^		−0.0031^***^		−0.0030^***^		−0.0029^***^
		(0.0007)		(0.0008)		(0.0007)		(0.0007)
*size*		−0.0212^***^		−0.0194^***^		−0.0218^***^		−0.0193^***^
		(0.0024)		(0.0025)		(0.0024)		(0.0024)
Time fixed effects	YES	YES	YES	YES	YES	YES	YES	YES
Firm fixed effects	YES	YES	YES	YES	YES	YES	YES	YES
*N*	56,153	55,405	48,960	48,285	56,153	55,405	56,153	55,405
*F*	198.4556	131.2926	169.9500	113.9049	194.6765	129.3505	129.3115	106.3745
*R*^2^-adj	0.0834	0.1132	0.0829	0.1124	0.0808	0.1112	0.0892	0.1185

*(1) Clustering robust standard errors in parentheses; (2)*

*** *p < 0.01*,

** *p < 0.05, and*

* *p < 0.1*.

In order to further explain the impact of tax sharing degree on the DVAR of firms, this study constructs the variable *Share Ratio*, based on the process of tax sharing reform. Specifically, in 2002, the proportion to share revenue from income taxes was 50% for the central government and 50% for the local government. However, since 2003, it has been 60% for the central government and 40% for the local government. We use the regression of continuous DID method to estimate the effect of tax sharing degree on the DVAR of firms. The results are shown in columns (5) and (6) in Table 2. It can be seen that the increase of tax share proportion significantly reduces the DVAR of firms.

According to the results of columns (1)–(6) in Table 2, the tax sharing reform not only significantly reduces the DVAR of firms but also has a stronger hindering effect on the DVAR of firms with the deepening of the reform. With the increase of tax sharing ratio, the reform has a greater obstacle to the export value-added rate of firms. In addition, the tax sharing reform may have a long-term effect on the DVAR of firms, while the model (1) only evaluates the average effect of tax sharing on the DVAR of firms. To examine its long-term effect, we perform the regression in the model (2). The results are shown in columns (7) and (8) in Table 2. It can be seen that the effect of tax sharing reform on DVAR is not significant in the current period. However, as the reform goes on, the hindering effect of tax sharing reform on the DVAR of firms enhances. For the control variables, firm productivity is an important factor to promote DVAR. Higher productivity can enable firms to maintain a strong competitive advantage in the market and help firms increase DVAR. However, firm size has a negative effect on DVAR, and it is significant at the level of 1%, indicating that small-scale firms tend to have a higher DVAR. The possible reason is that large-scale firms rely too much on the input of imported intermediate goods.

### Robustness Tests

The results in Table 2 show that tax sharing not only reduces the DVAR of firms, but that the hindering effect also enhances with the deepening of reform. Additionally, with the increase of the tax sharing ratio, the hindering effect on DVAR also enhances. To guarantee the validity and robustness of the regression results, a set of robustness tests is carried out from the perspectives of policy shock interference, firm innovation, variable replacement, and counterfactual test. The specific tests are as follows:

(1) Controlling the interference of other policies:

This study mainly investigates the impact of tax sharing on the DVAR of firms during the period of 2000–2006. However, the implementation of other macro policies in this period may affect the DVAR of firms, which influences the effect that we evaluate. To exclude the interference of relevant policies, this study focuses on the following policies:

- The accession of China to the WTO in 2001. Considering that the accession of China to the WTO at the end of 2001 has a great impact on the trade development of China, this historical event not only accelerates trade liberalization and opening up but also promotes the active participation of China in the global trade division and sharing of trade gains. Therefore, the accession of China to the WTO has a potential impact on the change of the DVAR of firms. To exclude its influence, this study has done the following two treatments: first, according to the time of the accession of China to the WTO, a dummy variable (*wto*) is generated. When the value of the variable is 1, it means that China has joined the WTO after 2001. Otherwise, it means that China has not joined the WTO. Second, since the accession of China to the WTO has more impact on “general trade,” it is necessary to exclude “general trade” from samples to reduce the impact of joining WTO on the results of this study. The specific regression results are shown in columns (1) and (2) in Table 3. It can be seen that the tax sharing reform still has a significant hindering effect on DVAR after removing the interference of WTO, and the results have not changed significantly. Additionally, from column (1), it can be seen that the accession to the WTO also promotes the DVAR of firms, and that the effect is significant at the level of 1%.
- The reform of the exchange rate system of China in 2005. China carried out the reform of RMB exchange rate system in 2005, which made the RMB exchange rate to revalue. Considering the influence of exchange rate appreciation, this study excludes the samples of 2005 and subsequent years from the regression, and only the samples of 2000–2004 are regressed. In this way, the influence of exchange rate system reform will be effectively eliminated. The specific results are shown in column (3) of Table 3. It can be seen that the elimination of the influence of RMB exchange rate reform does not change the hindering effect of tax sharing on the DVAR of firms, which further indicates the robustness of the above results.
- Value added tax reform in Northeast China in 2004. In addition, China has also carried out VAT reform in Northeast China. The specific process of the reform is as follows: from July 1, 2004, VAT transformation was first carried out in the equipment manufacturing industry, petrochemical industry, and eight other industries in three northeastern provinces; from July 1, 2007, the pilot scope was expanded to eight major industries, such as power industry and extractive industry in 26 old industrial base cities in six provinces in central China; since January 1, 2009, VAT reform has been carried out in all regions and industries in China. In order to avoid the bias caused by the VAT reform on the results of this study, we exclude the samples from the three northeast provinces in the regression. The specific results are shown in the column (4) of Table 3. With the elimination of the influence of VAT reform, tax sharing reform still significantly reduces the DVAR of firms.

**Table 3:** Robustness tests.

	**DVAR**	**DVAR**	**DVAR**	**DVAR**	**DVAR**	**DVAR**	**DVAR**	**DVAR10**	**DVAR**	**DVAR**	**DVAR**	**DVAR**
	**(1)**	**(2)**	**(3)**	**(4)**	**(5)**	**(6)**	**(7)**	**(8)**	**(9)**	**(10)**	**(11)**	**(12)**
*Share × Post*	−0.0187^***^	−0.0182^***^	−0.0178^***^	−0.0181^***^	−0.0187^***^	−0.0187^***^	−0.0168^***^	−0.0179^***^	−0.0187^***^	−0.0195^***^		
	(0.0023)	(0.0029)	(0.0027)	(0.0023)	(0.0023)	(0.0023)	(0.0031)	(0.0022)	(0.0023)	(0.0024)		
*wto*	0.0822^***^											
	(0.0038)											
*Tax*					−0.0023							
					(0.0020)							
*Tax ratio*						−0.0246^*^						
						(0.0146)						
*Innovation*									−0.0005			
									(0.0042)			
*random1*											−0.0008	
											(0.0012)	
*random2*												−0.0012
												(0.0011)
Control variables	YES	YES	YES	YES	YES	YES	YES	YES	YES	YES	YES	YES
Time fixed effects	YES	YES	YES	YES	YES	YES	YES	YES	YES	YES	YES	YES
Firm fixed effects	YES	YES	YES	YES	YES	YES	YES	YES	YES	YES	YES	YES
*N*	55,405	41,492	31,379	53,087	55,405	55,405	34,298	55,403	55,405	49,913	55,405	55,405
*F*	131.2926	132.2873	65.0496	127.3256	123.2498	123.3331	76.1551	147.9288	123.0955	121.5373	129.4160	129.4396
*R*^2^-adj	0.1132	0.1436	0.0783	0.1151	0.1133	0.1133	0.1121	0.1227	0.1132	0.1149	0.1111	0.1111

*(1) Clustering robust standard errors in parentheses; (2)*

*** *p < 0.01 and*

* *p < 0.1*.

(2) Export tax rebate:

In addition to the interference of macro policies, the implementation of the export tax rebate policy is also an important influencing factor that cannot be ignored. We eliminate this influencing factor by reviewing the policy documents in the period 2000–2006 about the export tax rebate, sorting out the tax rebate proportion regulated by different industries in different years, and using the change of the proportion to assign a value. Specifically, based on the above documents, this study processes the export tax rebate in the following three ways: First, generate a dummy variable *Tax* according to whether the industry carries out the export tax rebate reform. The value of this variable is 1, indicating that the industry has implemented export tax rebate, otherwise, it has not implemented export tax rebate; second, we assign a value to the industry according to the change in the proportion of export tax rebate, and generate variable *Tax ratio*, which represents the impact of export tax rebate on different industries. Third, all industries involved in the implementation of export tax rebate are excluded from the regression analysis to eliminate the interference of export tax rebate. The specific results are shown in columns (5)–(7) of Table 3. It can be seen that the regression coefficient has a little change compared with the results in column (2) of Table 2, which fully indicates that export tax rebate does not influence the effect of tax sharing on DVAR.

(3) Taking an alternative of indicator:

It is assumed that the share of foreign materials in domestic raw materials is 5% when we calculate the DVAR of firms. However, according to the research of Koopman et al. (2012), processing trade firms in China make the largest share of domestic raw materials and foreign products reach about 10%. The different proportions of foreign products will affect the calculation of the export value-added rate of firms, which will further influence the results of this study. In view of this, if we recalculate the DVAR of firms with the share of foreign materials in domestic raw materials accounting for 10%, we can obtain *DVAR10*. Then, the model (1) is regressed with *DVAR10* as the dependent variable. The results are shown in column (8) of Table 3. The results show that the tax sharing reform reduces the DVAR of firms, and that the effect is significant at the 1% level, indicating that the change of the share of foreign materials in domestic raw materials in the process of calculating DVAR does not influence the results of this article.

(4) Firm innovation:

Considering that the innovative activities of firms potentially affect the DVAR of firms, the above analysis does not effectively control them, which may affect the results of the above analysis. Therefore, we attempt to quantify the innovation activities of firms and directly put them into the model (1) to effectively control the influence of firm innovation. Based on the availability of data, this study uses the proportion of new product sales in total sales of firms (*Innovation*) to measure the innovation activities of firms. The specific results are shown in column (9) of Table 3. It can be seen from the results that after controlling the innovation activities of firms, the sign and significance of the regression coefficient of tax sharing on DVAR have not changed obviously, which indicates the robustness of the regression results.

(5) Excluding firms with age <6 years:

Moreover, the effect of tax sharing may have an impact on the establishment year and the strategic choice of tax avoidance, which may underestimate the effect of tax sharing on the DVAR of firms. To eliminate this influence, this study further excludes new firms established since the new century and firms with age <6 years. The results are shown in column (10) of Table 3. It is found that the tax sharing still significantly reduces DVAR after excluding the firms with age <6 years. The increase of the estimation coefficient indicates that the existence of new firms leads to the overestimation of the coefficient. However, in general, the exclusion of samples does not change the above conclusions, which show the validity of the results.

(6) Counterfactual tests:

Are the effects of tax sharing on the DVAR of firms affected by other random factors? To answer the question, this study performs regional counterfactual tests to exclude other influences. We examine the whole sample and the sample from the eastern region. We randomly select half of the firms in each group as the hypothetical intervention group for the tax sharing reform, and the other samples belong to the control group. We use the DID method to evaluate the effect of the hypothesis. If the effect is the same significant real effect, it indicates that there are still interferences from other factors. Otherwise, the effect is entirely from the tax sharing reform. The specific results are shown in columns (11) and (12) of Table 3. Column (11) reports the regression results of the intervention group randomly selected from the eastern region sample, and column (12) reports the regression results of the intervention group randomly selected in the overall sample. It can be seen that the same significant effect is not obtained, which indicates that there is no other random factor affecting the results. It further proves that the hindering effect on DVAR is not caused by other factors, but by the tax sharing reform.

(7) Sample selection bias due to ownership:

In the DID analysis, the sample of the intervention group is basically composed of local private firms, while the sample of the control group is mostly central firms and foreign-funded firms. Therefore, in addition to the above-mentioned factors that may cause evaluation bias, the differences in firm ownership and confounding factors caused by the differences, such as the higher level of technology owned by foreign-funded firms and the unequal status of state-owned firms and private firms in the domestic factor market, may also affect DVAR and the evaluation results. In view of this, this study tries to eliminate these factors, and the following three treatments have been done: first, we generate dummy variables for foreign-funded (*foreign*) and state-owned firms (*state*) according to the nature of the firm, and test whether the nature will affect DVAR. The results are shown in columns (1) and (2) of Table 4. The results show that the DVAR of foreign-funded firms is significantly higher than that of other types of firms; second, to further control it, we add dummy variables representing foreign-funded and state-owned firms in the model. The results are shown in columns (3) and (4) of Table 4, and we can see that the effective control of the nature of the firm has not changed the hindering effect of tax sharing on DVAR, and that the effect is still significant at the level of 1%; Third, we exclude state-owned firms and non-Hong Kong, Macao, and Taiwan foreign-invested firms to reduce the impact of firm nature on the control group. The results are shown in columns (5) and (6) of Table 4. It is found that the coefficients do not change significantly, which indicates that the nature of the firm does not change the results above. Finally, considering that the implementation of the Western Development Policy makes the tax borne by firms in the western region different from that in the eastern region, to eliminate the interference caused by tax incentives, we exclude the sample of firms in the western region. The results are shown in column (7). It can be seen that the elimination of the western samples does not change the conclusion that tax sharing significantly inhibits the DVAR of firms, which shows the robustness of the results in this study.

**Table 4:** Firm ownership and sample selection bias.

	**DVAR**	**DVAR**	**DVAR**	**DVAR**	**DVAR**	**DVAR**	**DVAR**
	**(1)**	**(2)**	**(3)**	**(4)**	**(5)**	**(6)**	**(7)**
*Share × Post*			−0.0195^***^	−0.0200^***^	−0.0204^***^	−0.0218^***^	−0.0187^***^
			(0.0030)	(0.0025)	(0.0025)	(0.0028)	(0.0024)
*foreign*	0.0109^***^		−0.0011				
	(0.0019)		(0.0025)				
*state*		0.0025		−0.0084^**^			
		(0.0033)		(0.0036)			
Control variables	YES	YES	YES	YES	YES	YES	YES
Time fixed effects	YES	YES	YES	YES	YES	YES	YES
Firm fixed effects	YES	YES	YES	YES	YES	YES	YES
*N*	55,405	55,405	55,405	55,405	53,298	39,864	53,977
*F*	129.9967	129.4526	123.1953	123.1693	129.5028	78.3598	130.5882
*R*^2^-adj	0.1120	0.1111	0.1132	0.1133	0.1172	0.1048	0.1154

*(1) Clustering robust standard errors in parentheses; (2)*

*** *p < 0.01 and*

** *p < 0.05*.

### Investigation of Macro Factors

In addition to the above factors influencing the DVAR of firms, the change of macro factors in the region and industry where the firms belong may also have an impact on the DVAR. The investigation of this issue is the main task of this part. We will start from the following two aspects to conduct a detailed investigation of macro factors:

First, for the general quantifiable macroeconomic factors, they are measured with indicators in this study. The specific approach is to match the macroeconomic factors at the provincial level with the firm data, directly incorporate these factors into the model, and then effectively control them so as to eliminate the impact of macroeconomic factors on the export value-added rate of firms. Combined with the contents of this study, we measure the level of economic growth with the logarithm of regional GDP, the degree of regional openness with the proportion of total foreign direct investment to GDP, the scale of regional government with the proportion of fiscal expenditure to GDP, the degree of regional financial development with the proportion of total regional loans to GDP, and the level of urbanization with the proportion of urban population to total population, and the regional cost is represented by the logarithm of regional total wages. The specific regression results are shown in columns (1)–(6) of Table 5. It can be seen that there is still a hindering effect of tax sharing on the DVAR of firms after the effective control of macroeconomic factors. Additionally, the increase of regional cost will not be conducive to the increase of DVAR. The local economic growth can improve the DVAR of firms, but the effect is not significant.

**Table 5:** Investigation of macro factors.

	**DVAR**	**DVAR**	**DVAR**	**DVAR**	**DVAR**	**DVAR**	**DVAR**	**DVAR**	**DVAR**	**DVAR**
	**(1)**	**(2)**	**(3)**	**(4)**	**(5)**	**(6)**	**(7)**	**(8)**	**(9)**	**(10)**
*Share × Post*	−0.0185^***^	−0.0150^***^	−0.0186^***^	−0.0186^***^	−0.0197^***^	−0.0176^***^	−0.0188^***^	−0.0173^***^	−0.0104^***^	−0.0101^***^
	−0.0023	−0.0024	−0.0023	−0.0023	−0.0024	−0.0023	−0.0023	−0.0023	−0.003	−0.003
*gdp*	0.0341									
	(0.0213)									
*open*		−0.0003^***^								
		(0.0000)								
*government*			−0.4727^***^							
			(0.0893)							
*financial*				−0.0124						
				(0.0112)						
*urban*					0.0224					
					(0.0299)					
*wage*						−0.1337^***^				
						(0.0183)				
Industry fixed effects	NO	NO	NO	NO	NO	NO	YES	YES	NO	NO
γ × *t*	NO	NO	NO	NO	NO	NO	NO	YES	NO	NO
γ × *t*^2^	NO	NO	NO	NO	NO	NO	NO	YES	NO	NO
City fixed effects	NO	NO	NO	NO	NO	NO	NO	NO	YES	YES
φ × *t*	NO	NO	NO	NO	NO	NO	NO	NO	NO	YES
φ × *t*^2^	NO	NO	NO	NO	NO	NO	NO	NO	NO	YES
Control variables	YES	YES	YES	YES	YES	YES	YES	YES	YES	YES
Time fixed effects	YES	YES	YES	YES	YES	YES	YES	YES	YES	YES
Firm fixed effects	YES	YES	YES	YES	YES	YES	YES	YES	YES	YES
*N*	55,405	53,728	55,405	55,358	47,851	55,358	55,405	55,405	41,806	41,806
*F*	123.1556	118.4254	124.6107	123.0763	94.5	123.9925				
*R*^2^-adj	0.1133	0.1155	0.1148	0.1133	0.1081	0.116	0.1139	0.1273	0.1378	0.1393

*(1) Clustering robust standard errors in parentheses; (2)*

*** *p < 0.01; (3) control variables, time fixed effects, and firm fixed effects are added to the above regressions. Due to space limitations, they are not presented in the table here; (4) since there may be mobility among firms, we have joined the firm, industry and city FEs here at the same time*.

Second, for the unobservable factors in the region where the firm is located and the industry it belongs to, this study controls these factors by incorporating regional fixed effects and industry fixed effects into the model, and considers the regional non-linear trends and industry non-linear trends. Another purpose of this approach is to test the parallel trend hypothesis required by the DID. This study takes the tax sharing reform that began in 2002 as the quasi-natural experiment, and the investigation period is limited to 2000–2006. Therefore, there is only the two-period data before the implementation of the tax sharing reform. The few years before the implementation of this policy makes it unable to a perform regression test on the parallel trend hypothesis. Therefore, following Moser and Voena (2012), the fixed effects of regions and industries and their non-linear trends are added to the model. The specific regression results are shown in columns (7)–(10) of Table 5. Columns (7) and (8) are mainly for the analysis of industry fixed effects and non-linear trends, while columns (9) and (10) are for the investigation of regional fixed effects and non-linear trends. The results show that after controlling the regional and industry effects and their non-linear trends the tax sharing reform reduces the DVAR of firms, and that the effect is still significant at the level of 1%. From the perspective of regression coefficients, there is no large fluctuation in the coefficients after controlling the industry. However, the control of regional fixed effects makes the evaluation coefficient fluctuate greatly. In general, the control of macro factors that are difficult to quantify in the regions and industries has not changed the hindering effect of tax sharing on the DVAR of firms. It also shows that the parallel trend assumption required by the DID method is satisfied in this study, which further proves the robustness of the conclusions of this paper.

## Influencing Mechanism

In this section, we will focus on how fiscal centralization influences the DVAR of firms. Through the theoretical elaboration in the second section, it can be seen that there may be two types of potential paths for tax sharing to have an impact on the DVAR of firms. One is the role of the cost markup rate; the other is the change of the price of intermediate goods input. Therefore, we will try to analyze these two mechanisms and estimate their impact on DVAR.

### Calculation of Cost Markup Rate

As the core of the mechanism analysis in this study, the calculation of the cost markup rate is another major task. According to the definition of the cost markup rate of the firm, it is necessary to know the price and marginal cost of the products of the firm in order to measure it. However, from the data used in this study, the China Industry Business Performance Database does not directly give product prices and marginal costs. In view of this, De Loecker and Warzynski (2012) use the cost minimization method and express the cost markup rate of the firm as the ratio of the output elasticity of the intermediate to the expenditure share of the element. The new method of calculating cost markup rate is specifically expressed as follows:


(7) $$\begin{array}{l} \mu_{it}=\theta_{it}^{x}{\left(\alpha_{it}^{x}\right)}^{-1} \end{array}$$

where μ_*it*_ represents the cost markup rate, $\theta_{it}^{x}$ represents the output elasticity of intermediate input *x*, and $\alpha_{it}^{x}$ represents the ratio of intermediate input *x* to the expenditure.

For the calculation of the above formula, this study adopts the structural model by Xu and Li (2018) to deal with the unobservable productivity shocks and price factors, which will not require to consider the factors of demand structure. It can be seen from the China Industry Business Performance Database that the proportion of intermediate input expenditure can be obtained directly from the firm-level data. Therefore, the key to calculate the cost markup rate is the unbiased estimation of the output elasticity of the intermediate input, which needs the unobservable productivity shocks to be controlled. Therefore, this study uses the two-step method to make a robust estimation of the cost markup rate of the firm, and this method can deal with the endogenous problem of productivity. For the form of the production function, the translog production function is used here. The specific form is as follows:


(8) $$\begin{array}{l} y_{it}=\beta_{l}l_{it}+\beta_{k}k_{it}+\beta_{x}x_{it}+\beta_{ll}l_{it}^{2}+\beta_{kk}k_{it}^{2}+\beta_{xx}x_{it}^{2}+\beta_{lk}l_{it}k_{it}\\ +\beta_{kx}k_{it}x_{it}+\beta_{lx}l_{it}x_{it}+\beta_{lkx}l_{it}k_{it}x_{it}+\omega_{it}+\varepsilon_{it} \end{array}$$

where *y* represents the total industrial output. *k, l*, and *x* represent the capital input, labor input, and intermediate input, respectively. All indicators are converted to constant price and logarithmic value. ω represents the heterogeneous productivity of the firm. ε is a random error term. The first step in the two-step method is to estimate the output *y*_*it*_ of the firm and obtain its unbiased estimation ${\hat{\phi }}_{it}$, and use a non-parametric method to obtain the random productivity shock ${\hat{v}}_{it}\left(\beta \right)$. According to the initial decision of firm capital *k*, and if the nature of labor *l* and intermediate input *x* are not related to the productivity of the lagging one-stage period, the following moment condition are obtained:


(9) $$\begin{array}{l} E[{\hat{v}}_{it}(\beta )(l_{i,t-1},k_{it},x_{i,t-1},l_{i,t-1}^{2},k_{it}^{2},x_{i,t-1}^{2},\\ l_{i,t-1}k_{it},k_{it}x_{i,t-1},l_{i,t-1}x_{i,t-1},l_{i,t-1}k_{it}x_{it}{)}^{\prime }]=0 \end{array}$$

For the above equation, this study uses the generalized moment estimation method to estimate all the parameters in the production function, and then obtain the output elasticity of the intermediate input of the firm${\hat{\beta }}_{x}+2{\hat{\beta }}_{xx}x_{it}+{\hat{\beta }}_{lx}l_{it}+{\hat{\beta }}_{kx}k_{it}+{\hat{\beta }}_{lxk}l_{it}k_{it}$. Finally, the estimation of cost markup rate $\hat{\mu }$ can be calculated.

### Analysis of Influence Mechanism

If firms are faced with local partiality and tax competition under tax sharing, they will reduce production and operation costs to a large extent driven by tax evasion incentives. Therefore, under the circumstances of prominent export advantages, the export industry with excess profit attracts more firms to participate in, which will be reflected in the linear rise of cost markup rate (Berman et al., 2012; Yu and Cui, 2018). For individual firms, the cost markup rate reflects the ratio of total output to total investment. Under the condition that the output value remains unchanged in a short time, the reduction of input cost will inevitably promote the increase of profit rate (Mao and Xu, 2018). With the increase of local tax sharing under fiscal centralization, the increase of the cost markup rate of private firms will lead to the corresponding increase of DVAR. However, the tax competition of local governments aims to attract firms to enter, improve business performance, and make up for the tax reduction deficit. Therefore, the export firms whose main business is the export of products and services will have to bear more export pressure and responsibility after local tax sharing. In this case, in order to achieve the economic performance set by the local government, the downstream producer will increase their export efforts, so as to increase the demand for intermediate goods in the market in a short time, and it will further raise the price of domestic intermediate goods. Compared with the constant price of imported intermediate goods, the increase of the price of domestic intermediate goods will reduce the relative price of imported intermediate goods. Furthermore, it has a crowding-out effect on the market input of domestic intermediate goods, which will inhibit the DVAR of firms.

To test whether the above two mechanisms hold or not, we choose the firm cost markup rate and the relative price of imported intermediate goods as intermediary variables and conduct an empirical test. The specific intermediary effect models are as follows:


(10) $$\begin{array}{l} Middle_{it}=\theta_{1}+\theta_{2}Share_{i}\times Post_{t}+\sum Control_{it}+\mu_{i}+\delta_{t}\\ \;+\varepsilon_{it} \end{array}$$

(11) $$\begin{array}{l} Mkp_{it}=\varphi_{1}+\varphi_{2}Share_{i}\times Post_{t}+\sum Control_{it}+\mu_{i}+\delta_{t}\\ \;+\varepsilon_{it} \end{array}$$

(12) $$\begin{array}{l} DVAR_{it}=\phi_{1}+\phi_{2}Share_{i}\times Post_{t}+\eta Mkp_{it}+\sum Control_{it}\\ \;+\mu_{i}+\delta_{t}+\varepsilon_{it} \end{array}$$

(13) $$\begin{array}{l} DVAR_{it}=\gamma_{1}+\gamma_{2}share_{i}\times Post_{t}+\rho Middle_{it}+\sum Control_{it}\\ \;+\mu_{i}+\delta_{t}+\varepsilon_{it} \end{array}$$

where *Middle*_*it*_ represents the intermediate goods input in firm *i* in year *t*. *Mkp*_*it*_ represents the cost markup rate of firm *i* in year t. Models (10) and (11) mainly investigate the effect of tax sharing on the input of intermediate goods and cost markup rate of firms. While models (12) and (13) mainly analyze the effect of tax sharing on DVAR when the cost markup rate and relative price of imported intermediate goods are considered as intermediary variables.

Based on the above regression, the test results are shown in Table 6. Columns (1) and (2), respectively, regress the firm cost markup rate and the import intermediate goods input to the DVAR. The results show that they both have a significant effect on DVAR at the level of 1%, but that the effect is opposite. Then, we test the effect of tax sharing on foreign intermediate good input and firm cost markup rate in columns (3) and (5). It can be found that, as the theoretical analysis shows, on the one hand, the tax sharing reform improves the profit of firms, thus promoting the cost markup rate. On the other hand, it also improves the market price of domestic intermediate goods and further increases the total amount of imported intermediate goods. Therefore, are these two mechanisms established? In column (4), the intermediary variable of imported intermediate good input is added. The result shows that this variable has a significant negative effect on the DVAR of firms in the regression model, indicating that the centralized tax reduces the relative price of imported intermediate goods, and that it has a significant crowding-out effect on domestic intermediate goods. Under the situation of domestic intermediate market declining, the DVAR of firms is also restrained, and this mechanism can be established. In terms of cost markup rate, it is added as an intermediary variable in column (6). The effect of cost markup rate on the DVAR is no longer significant, and the coefficient has a great change (from 0.0528 to −0.0312), indicating the mechanism that cost markup rate effect in tax sharing has a significant effect on the DVAR is not established. Therefore, this mechanism cannot be used as the real logic of the centralized tax system to improve DVAR.

**Table 6:** Influence mechanism tests.

	**DVAR**	**DVAR**	**Middle**	**DVAR**	**Mkp**	**DVAR**
	**(1)**	**(2)**	**(3)**	**(4)**	**(5)**	**(6)**
*Share × Post*			0.0615^***^	−0.0109^***^	0.0081^***^	−0.0103^***^
			(0.0132)	(0.0017)	(0.0013)	(0.0030)
*Mkp*	0.1751^***^	0.0528^***^				−0.0312
	(0.0198)	(0.0193)				(0.0193)
*Middle*	−0.1150^***^	−0.1385^***^		−0.1270^***^		
	(0.0016)	(0.0017)		(0.0014)		
Control variables	No	YES	YES	YES	YES	YES
Time fixed effects	YES	YES	YES	YES	YES	YES
Firm fixed effects	YES	YES	YES	YES	YES	YES
*N*	42,449	41,804	55,405	55,405	41,804	41,804
*F*	818.656	526.0692	294.9704	580.0916	643.7168	122.8412
*R*^2^-adj	0.4383	0.5312	0.2291	0.5065	0.4609	0.137

*(1) Clustering robust standard errors in parentheses; (2)*

*** *p < 0.01*.

The above mechanism analysis provides further evidence for the results of this study. In the theoretical analysis, on the one hand, we think that the cost markup effect can improve DVAR. On the other hand, we think that the crowding-out effect of intermediate goods can inhibit the increase of VAR. Through the mechanism tests, we find that the promotion effect is not significant, but that the hindering effect is significant, which further provides an empirical basis for the negative impact of fiscal centralization on DVAR.

## Conclusions

Taking the implementation of income tax sharing reform in China in 2002 as a quasi-natural experiment, this study examines the effect of fiscal centralization on DVAR by the DID method. It is found that with the further tightening of the fiscal power of the central government, the grab for local income tax may significantly reduce DVAR. For local governments, the strategy to transfer the tax burden to local firms often works in the short term, but this strategy is not sustainable in a long-term development. This short-sighted behavior of replacing “quality improvement” with “quantity increase” is not conducive to the improvement of the export competitiveness and enhancement of sustainable export innovation behavior of firms, thus hindering the climbing of firms in the global value chain.

The above conclusions have very important policy implications for deepening the reform of the tax system and promoting the rise of the global value chain of export firms. Specifically: (1) reasonable sharing system arrangements and the standard behaviors of local governments are of great importance to cultivating a good export environment for firms. Therefore, it is necessary to continue to deepen the reform of income tax sharing and maximize the neutral principle and incentive effect of taxation, so as to provide necessary institutional advantages for promoting the DVAR of the export of Chinese firms and the construction of their own export competitiveness. (2) Since the competitive crowding-out effects of processing trade firms with different output elasticity of intermediate products are different, appropriate tax incentives or government subsidies can be given to firms according to the characteristics of different types of firms. This helps firms improve their ability to promote DVAR, strive for more trade benefits in export competition, and form an export competitive advantage, so as to achieve a high-end position in the global value chain.

## Data Availability Statement

The data used to support the findings of this study have not been made available due to the third party restrictions. Requests to access these datasets should be directed to http://www.stats.gov.cn.

## Author Contributions

CF, BS, and CB: conceptualization. CF and BS: methodology. CF, BS, and SY: data. CF, BS, and HY: writing and original draft preparation. CF, SY, and CB: writing, review, and editing. HY and SY: investigation. BS and SY: funding acquisition. All authors contributed to the article and approved the submitted version.

## Funding

This study was supported by the Humanities and Social Science Fund of the Ministry of Education in China (Grant No: 20YJC790165), the National Social Science Foundation of China (Grant No: 20CGL005), China-Central Eastern European Countries Higher Joint Education Project (Grant No: 202028), and Shaanxi Provincial Natural Science Basic research program (Grant No: 2021JQ-457).

## Conflict of Interest

The authors declare that the research was conducted in the absence of any commercial or financial relationships that could be construed as a potential conflict of interest.

## Publisher's Note

All claims expressed in this article are solely those of the authors and do not necessarily represent those of their affiliated organizations, or those of the publisher, the editors and the reviewers. Any product that may be evaluated in this article, or claim that may be made by its manufacturer, is not guaranteed or endorsed by the publisher.

## Footnotes

^1^We assume that the foreign content share in domestic raw material is 10% in the following robustness tests.

^2^Over importing firms are defined as firms whose actual volume of imported intermediate goods is more than the sum volume of intermediate input.

^3^The mixed trade firm is a firm that carries out general trade and processing trade at the same time.

^4^Table 1 only reports the descriptive statistics of the initial data.
